# Predictive Analytical Modeling of Thermo-Mechanical Effects in Orthogonal Machining

**DOI:** 10.3390/ma14247876

**Published:** 2021-12-19

**Authors:** Alliche Mohamed-Amine, Djennane Mohamed, Djebara Abdelhakim, Songmene Victor

**Affiliations:** 1 Laboratoire de Mécanique et Systèmes Énergétiques Avancés (LMSEA), Department of Mechanical Engineering, École Nationale Polytechnique de Constantine, Constantine 25000, Algeria; alliche.k.amine@gmail.com (A.M.-A.); djenane_m@yahoo.fr (D.M.); 2 Laboratoire D’Ingénieriedes Produits, Procédés et Systèmes(LIPPS), Departments of Mechanical Engineering, École de Technologie Supérieure, Montreal, QC H3C 1K3, Canada; victor.songmene@etsmtl.ca

**Keywords:** Johnson-Cook model, dry machining, cutting temperature, dust generation, RSM

## Abstract

Factor relationships in a machining system do not work in pairs. Varying the cutting parameters, materials machined, or volumes produced will influence many machining characteristics. For this reason, we are attempting to better understand the effect of the Johnson-Cook (J-C) law of behavior on cutting temperature prediction. Thus, the objective of the present study is to investigate, experimentally and theoretically, the tool/material interactions and their effects on dust emission during orthogonal cutting. The proposed approach is built on three steps. First, we established an experimental design to analyze, experimentally, the cutting conditions effects on the cutting temperature under dry condition. The empirical model which is based on the response surface methodology was used to generate a large amount of data depending on the machining conditions. Through this step, we were able to analyze the sensitivity of the cutting temperature to different cutting parameters. It was found that cutting speed, tool tip radius, rake angle, and the interaction between the cutting speed and the rake angle explain more than 84.66% of the cutting temperature variation. The cutting temperature will be considered as a reference to validate the analytical model. Hence, a temperature prediction model is important as a second step. The modeling of orthogonal machining using the J-C plasticity model showed a good correlation between the predicted cutting temperature and that obtained by the proposed empirical model. The calculated deviations for the different cutting conditions tested are relatively acceptable (with a less than 10% error). Finally, the established analytical model was then applied to the machining processes in order to optimize the cutting parameters and, at the same time, minimize the generated dust. The evaluation of the dust generation revealed that the dust emission is closely related to the variation of the cutting temperature. We also noticed that the dust generation can indicate different phenomena of fine and ultrafine particles generation during the cutting process, related to the heat source or temperature during orthogonal machining. Finally, the effective strategy to limit dust emissions at the source is to avoid the critical temperature zone. For this purpose, the two-sided values can be seen as combinations to limit dust emissions at the source.

## 1. Introduction

The machining process is an operation to remove material from the workpiece in the form of chips. The cutting forces imposed by the tool converts the mechanical energy into plastic deformation of the material, which impacts the machining performance by generating different chip morphologies [1]. This plastic deformation generates a shear plane which is characterized by the shear angle that mainly influences the chip formation mechanism [2,3,4]. In addition, the presence of friction at the tool/chip interface is considered an unfavorable condition for machining and induces undesirable thermal effects. To reduce the friction effects, various lubricants are introduced at the tool/chip interface during machining [5,6]. Proper cooling techniques (high pressure cooling, minimum quantity lubrication, or cryogenic cooling) should be adopted to reduce the cutting temperature effect [7,8,9]. The mechanics of machining chip formation depend on the control of cutting parameters and tool geometry [10]. This dependence directly indicates the frictional behavior at the tool/chip interaction points [11].Many experimental investigations related to chip formation have focused on shear angle, chip segmentation and specific cutting force, and they have found that the cutting temperature predominantly affects the chip formation characteristics. Mia et al. used a multi-objective optimization of some parameters representative of the tool/chip interaction using the Taguchi method [12]. One of the objectives of this optimization is to minimize the cutting temperature and maximize the shear angle. Analysis revealed that the cutting speed and feed rate exert the most dominant effect on both responses. Gupta et al. conducted experiments based on the response surface methodology [13]. Their results show that metal cutting operations, proper selection of machining parameters and cutting fluids affect the cutting mechanism, which is associated with cutting forces, tool wear, cutting temperature and surface finish of the machined part. The studies carried out by these researchers are focused on the machining results that they are directly interested in. These desired parameters are surface quality, tool life and wear, cutting force and temperature.

Dry machining has been used for the removal of cutting fluids [14]. This is to avoid the environmental, health and safety issues associated with wet machining [15,16,17,18,19,20]. For some materials, dry machining generates dust. Therefore, dry machining should also be given special attention from an air quality perspective. Ugulino and Hernández developed a regression model to estimate dust generation for each size fraction as a function of the average chip thickness and depth of cut [21]. They concluded that independent cutting parameters can influence dust generation. Khettabi et al. developed a hybrid model of particle generation during machining processes that was based on the Needelman-Lemonds constitutive equations, combined with tool-chip friction and plastic deformation of materials [22]. Djebara et al. evaluated the dust generation based on the Johnson-Cook equations combined with the Oxley equations of the same model proposed by Khettabi [22,23]. Their results show an excellent fit compared to the model based on Needelman-Lemonds equations. In addition, good agreement was found between the predicted and measured dust generation for the different materials tested. Sutherland et al. reported that the aerosol concentration during dry machining was much lower than that produced by wet machining operations [24]. Research results showed that speed, feed rate and depth of cut were key variables in dust formation during cast iron machining. Khettabi et al. deduced that plastic deformation should be controlled to reduce the generation of particles during the machining process [7]. Songmene et al. concluded that the formation of fine and ultrafine particles during machining is attributable to different phenomena (macroscopic and microscopic friction, plastic deformation and mode of chip formation) [25]. Djebara et al. studied the effect of artificial aging on machinability using an innovative criterion, which is dust generation [26]. Their results indicate that the artificial aging involved in the cutting process alters the mechanical properties of the material, and modifies the mode of chip formation and dust generation. However, the evaluation of dust generation as a function of the cutting temperature variation has never been addressed in researches.

Some constitutive relationships have been proposed to model the plastic flow of metallic materials. These analytical behavior models allow for approximating the material behavior as closely as possible by calculating and using constants for the material studied. The thermo-mechanical behavior of a material is represented by a mathematical expression that relates quantities, such as stress, strain, strain rate, and temperature of the machined part [27]. Semi-physical models use more physical observations, especially by making assumptions about the behavior of the deformation mechanisms involved [28,29]. Physical formalisms have a priori a higher predictive power than other formalisms because they have the particularity to take into account memory effects in the restitution of the mechanical behavior of the material. However, given the inconvenience of determining the many factors of the models and the difficulty of implementing these models in computational codes, we will not use these behavior laws in this work. Empirical models are derived from the phenomenological analysis used to interpret experimental data. For example, Needelman-Lemonds incorporated the effects of material temperature sensitivity and thermal conduction into an infinite band analysis of shear localization [30]. The Needelman-Lemonds constitutive equations can be used to estimate cutting forces and temperature. In addition, Johnson-Cook proposes an empirical law designed from experimental results for rapid implementation in computational codes [27,31]. This model is based on Ludwik’s model and includes the influences of strain rate, strain hardening and temperature [32]. The phenomenological approach chosen for the development of this model leads to decoupling the effects of the strain rate, temperature and work hardening on the flow stress. However, experimental tests are still required to acquire material factors under different machining conditions [33,34].

In light of the above facts, it is evident that researchers have studied how the prediction of cutting forces and temperature is necessary due to the difficulty and inaccuracy of experimental measurements when machining different materials. In this research we opted for the development of a model for the prediction of the cutting temperature. The model is based on a material behavior law that takes into account the effect of strain, strain rate and temperature. Several assumptions must be taken into account in order to incorporate the friction effect in the multi-physics problem, as material behavior, friction and temperature are all related to each other. However, the intermediate effects (cutting condition, geometrical parameters and friction) must also be studied with a direct focus. These intermediate effects will be optimized simultaneously using the response surface method. Finally, the most appropriate parameters and cutting environment will be suggested. The objective of this study will then be to investigate, experimentally and theoretically, the tool/chip interactions and the causal links between cutting temperature and dust generation during orthogonal cutting. For this purpose, we have chosen a J-C model to describe the behavior of the studied material and to better understand the effect of the J-C behavior law on the prediction of cutting parameters, such as the cutting temperature.

## 2. Materials and Methods

### 2.1. Experimental Procedures

**Figure 1:**
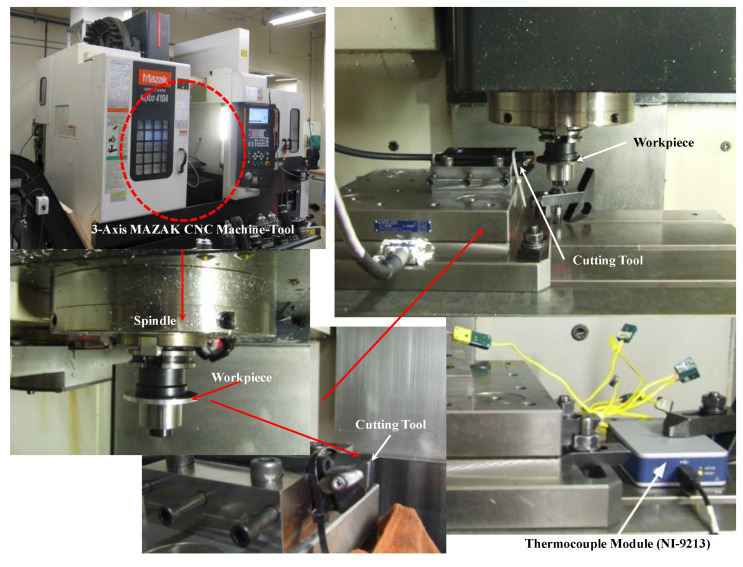
Experimental configuration of the orthogonal cutting.

Tests were carried out to determine the effect of setting factors (cutting speed, feed rate, nose radius tool, rake angle and clearance angle) on the evolution of the tool/chip cutting temperature. Orthogonal and dry machining tests were performed on a MAZAK three-axis CNC machine tool (Mazak iSMART Factory, Florence, KY, USA), using an uncoated carbide insert (TPGN-160308, Kennametal grade K68) (Kennametal Inc, pittsburgh, PA, USA). Disc-shaped parts with an outer diameter of 70 mm and a thickness of 4 mm were used in the experiment. The cutting width (4.3 mm) was kept constant for all experiments. The cutting temperature at the tool is measured using a chromel/alumel thermocouple (type K) (Pyromation, Fort Wayne, IN, USA) with a diameter of 0.075 mm. This technique is relatively easy to apply and involves measuring the average temperature over the whole tool/chip interface. In addition, a fine blind hole with a diameter of 0.9 mm was made in the cutting insert using an electric discharge machine (EDM) (Kent Industrial USA, INC, Tustin, CA, USA). The thermocouple was then inserted inside the wafer and the other end was connected to a data acquisition device (thermocouple module model NI-9213) (National Instruments, Austin, TX, USA). The response time of this thermocouple is 0.03 s. The uncertainty in the temperature measurement resulting from this type of thermocouple is 0.4% of the maximum value. The experimental configuration is schematically represented in Figure 1.

The chemical composition, physical properties, and Johnson-Cook model parameters for the orthogonal cutting of AMS-6414 steel are defined in Table 1 [35]:

### 2.2. Response Surface Methodology Implemented

The response surface methodology (RSM) has been established to determine the effects of each factor and the interactions between them, which allows for the optimization of efficient machining processes. In order to minimize the experimental work, the number of trials of this type of design were significantly reduced, while maintaining good accuracy. The proposed approach is based on an RSM experimental design to experimentally analyze the cutting temperature during dry machining. The cutting conditions were chosen while maintaining a response surface design with a total of 27 experiments generated according to the different combinations of the elements of cutting speed (*V*), feed rate (*f*), tool nose radius (*r_β_*), rake angle (*α*) and clearance angle (*γ*) during AMS-6414 orthogonal machining. This is completed with an analysis of variance of the difference factors tested. The factors to be studied and the respective levels are given in Table 2.

### 2.3. Theoretical Evaluation of the Cutting Temperature

Materials machining is a thermo-mechanical process and the temperature information in the chip formation zone is fundamental to identify the phenomenon. The Johnson-Cook model predicts the stress flux of materials subjected to high stresses, high strain rates and high temperatures [36]. The first term of the J-C model represents strain hardening, the second term expresses the strain rate effect and the third term represents the temperature effect. The temperature effect at the tool/chip interface is taken into account in this extension of the model. The evaluation of the temperature distribution along the tool/chip contact will be developed and studied. A comparison between the prediction model and the related experimental results will also be established.

The improvement made in the estimation of the value of the shear angle θ has been reincarnated into the mean friction angle term:

(1) $$\theta =A+\frac{\alpha -\lambda }{2},$$

*A* is a constant that depends on the material specification, experimental work has been done to predict the value of the angle *A*, in the case of steel and hard aluminum, it can be taken as *A* = 35°. The rake angle is *α*, and *λ* is the mean friction angle, which equals to atan (*μ*), and *μ* is the friction coefficient.

**Figure 2:**
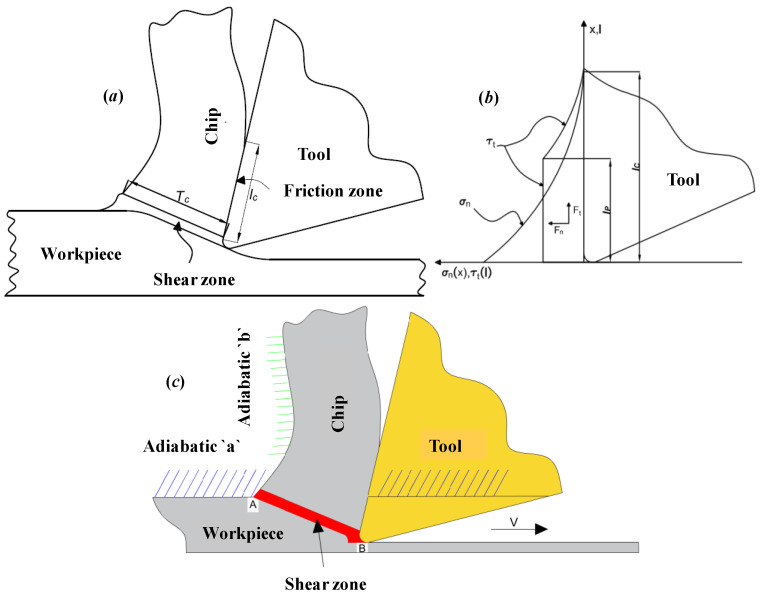
Tool/chip contact zone: (**a**) Heat source areas, (**b**) Normal and frictional stress distributions on the tool face and (**c**) Boundary effect integration in the heat distribution modeling [35].

The modeling of the frictional behavior under high strain rates and temperatures at the tool/chip interface is not fully understood. The next step in analytical modeling was to improve some features that were neglected or simplified in the model proposed by Djebara et al. [23]. This model assumes that the friction along the chip interface of the cutting tool is characterized by a constant friction coefficient. On the other hand, Zorev proposed that the contact area on the tool cutting face should be divided into two parts (Figure 2): the sticking region and the sliding region [35]. This model aims to measure the normal and shear stress distributions. In the sticking region, the shear stress is equal to the shear strength of the machined material and in the sliding region; the friction coefficient is independent of the normal stress. This model has been widely cited and verified by experimental investigations in previous studies, including some with a split tool measuring the normal pressure and shear stress distributions on the tool cutting face [37]. This model is characterized by the following equations:

(2) $$\tau_{friction}=\{\begin{array}{cc} \tau_{c} & 0\leq x\leq l_{p}\\ \mu_{local}\times P & l_{p}\leq x\leq l_{c} \end{array},$$

The experimental affinity confirms that the local friction coefficient increases with increasing temperature at the tool/chip interface and depends on the tool and workpiece properties. The normal pressure distribution is not uniform, but is a decreasing function of length contact at the tool/chip interface [38,39]. The pressure distribution is given as follows:

(3) $$P\left(x\right)=P_{0}{\left(1-\frac{x}{l_{c}}\right)}^{\iota },$$

where *P*_0_ is the normal stress on the cutting face at the tooltip and *i* is an exponential constant, which represents the pressure distribution. Bahi et al. found an expression for the normal stress *P*_0_ taking into account the chip equilibrium [37]:

(4) $$P_{0}=4\tau_{s}\left(\frac{i+1}{i+2}\right)\left(\frac{\cos\lambda^{2}}{\sin2\theta }\right),$$

A high strain rate in the material involves a high heat release which results in the thermo-mechanical coupling effect whereby part of the mechanical work is evacuated from the material as heat. Plastic work can be expressed as a function of the flow stress, strain and thermal variation, which can be expressed as follows [40]:

(5) $$\Delta T=\frac{\chi \sigma }{\rho \times c_{w}}{\dot{\varepsilon }}_{p},$$

By replacing this expression in the J-C formulation, it can be written as follows:

(6) $$\Delta T=\frac{\chi }{\rho \times c_{w}}{\dot{\varepsilon }}_{p}\left(A+B{\left(\varepsilon_{p}\right)}^{n}\right)\left(1+C\ln\left(\frac{{\dot{\varepsilon }}_{p}}{{\dot{\varepsilon }}_{0}}\right)\right)\left(1-{\left(\frac{T-T_{0}}{T_{m}-T_{0}}\right)}^{m}\right),$$

The flow stress can be expected using Von-Mises criteria:

(7) $$K_{AB}=\frac{1}{\sqrt{3}}\left(A+B{\left(\varepsilon_{p}\right)}^{n}\right)\left(1+C\ln\left(\frac{{\dot{\varepsilon }}_{p}}{{\dot{\varepsilon }}_{0}}\right)\right)\left(1-{\left(\frac{T-T_{0}}{T_{m}-T_{0}}\right)}^{m}\right),$$

The average temperature in the primary shear zone can be written as a function of the flow stress and the variable ψ(i) as follows:

(8) $$\Delta T=\psi (i)\times K_{AB},$$

The variable ψ(i) is expressed as:

(9) $$\psi (i)=\frac{\left(1-\xi \right)\cos\alpha }{\rho_{w}\times c_{w}\sin\phi \cos\left(\phi -\alpha \right)},$$

Integrating Equation (9) into Equation (8) gives:

(10) $$K_{AB}=\frac{1}{\sqrt{3}}\left(A+B{\left(\varepsilon_{p}\right)}^{n}\right)\left(1+C\ln\left(\frac{{\dot{\varepsilon }}_{p}}{{\dot{\varepsilon }}_{0}}\right)\right)\left(1-{\left(\frac{\psi (i)\times K_{AB}}{T_{m}-T_{0}}\right)}^{m}\right),$$

Solving Equation (10) to find the flow stress *K_AB_* and replacing its value Equation (8) leads to finding the temperature variation Δ*T*. The value of Δ*T* is selected by the minimum value between the results of Equations (5) and (6). The temperature in the primary shear zone *T_AB_* is given as a function of the ambient temperature, the temperature variation and by the Taylor-Quinney coefficient as follows:

(11) $$T_{AB}=T_{0}+\chi \Delta T,$$

In the machining process, a large part of the plastic work *W_pl_* is converted into thermal energy *Q_t_* due to internal friction which depends on the material. The Taylor-Quinney coefficient χ defines the percentage of plastic work which is adiabatically converted into heat from the plastic strain energy density [40].

(12) $$\chi =\frac{Q_{t}}{W_{pl}}=\frac{m\times c_{t}\times \Delta T}{W_{pl}},$$

The temperature in the secondary shear zone T_int_ is also specified as a function of the ambient temperature *T*_0_, the temperature variation Δ*T* and the maximum temperature increase in the shear zone *T_M_* by the variable factor *ψ* which can diverge between 0 ≤ *ψ* ≤ 1:

(13) $$T_{\mathrm{int}}=T_{0}+\Delta T+\psi T_{M},$$

Finally, Jaeger developed the theory of moving sources to express the maximum temperature, called spark temperature, reached at the surface of two semi-infinite solids in friction contact [41]. This physical phenomenon will be incorporated into our analytical model with two modifications to the classical moving band for the chip and stationary for the tool for application of orthogonal metal cutting. The total temperature increase at any point *M*(*x*,*z*) caused by the moving interface frictional heat source is given by:

(14) TAB(M)=12πλc∫−li=0+lQsheareX×V2acK0(R×VC2ac)dli,

where $Q_{\mathrm{shear}}$ is the heat total released from the shear zone which can be expressed as follows:

(15) $$Q_{\mathrm{shear}}=K_{AB}V_{S},$$

When the temperature variation of the tool/chip interface can be expected as:

(16) Tint(M)=1πλc∫li+lQfrictionB(li)e−(X−li)×VC2ac[K0(Ri×VC2ac)+K0(Ri×VC2ac)]dli,

$Q_{\mathrm{friction}}$ is the heat quantity generated by the shear zone, which can be expressed as follows:

(17) $$Q_{\mathrm{friction}}=K_{\mathrm{int}}\times c_{\mathrm{int}},$$

Many additions to the first model proposed by Djebara et al. have been reported [23]. In addition, the cutting forces due to the action of the tool on the workpiece for a given thickness and cutting speed are easily booked (flow stress, shear force, resultant force, cutting force, and feed force).

### 2.4. Model Processing

**Figure 3:**
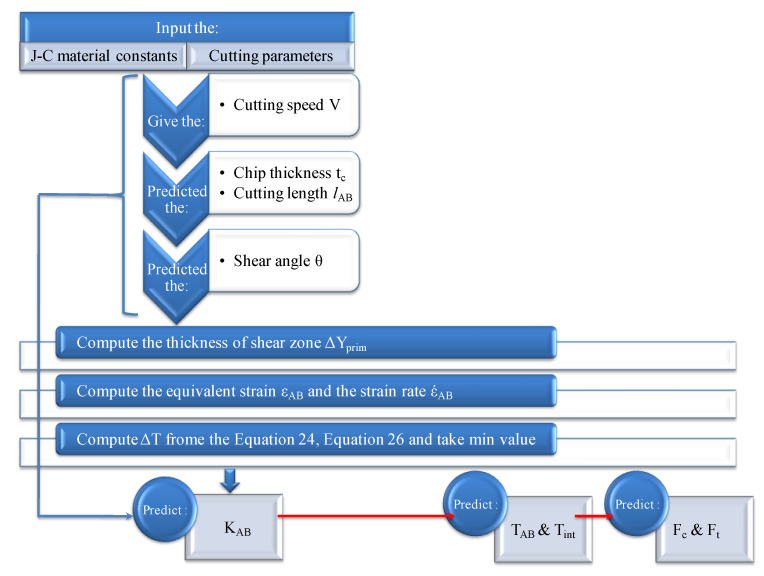
Flowchart for simultaneous temperature estimation in primary and secondary shear zones.

Based on the J-C model, the MATLAB script is defined according to the flowchart shown in Figure 3. The input was a definition of the material properties, cutting conditions and tool geometry. The compilation is preceded by the calculation of the shear angle and the chip geometry parameters. The increment of the cutting speed is then used to solve the flow stress equation in order to estimate the temperature value in the primary shear zone and in the secondary shear zone:

## 3. Results and Discussion

### 3.1. Experimental Design and Results

The temperature values obtained are in the range of 708 to 1012 °C. These results will be used in the following section to construct an empirical model of the tool/chip contact temperature.

#### 3.1.1. Direct Effects of Factors on Response

**Figure 4:**
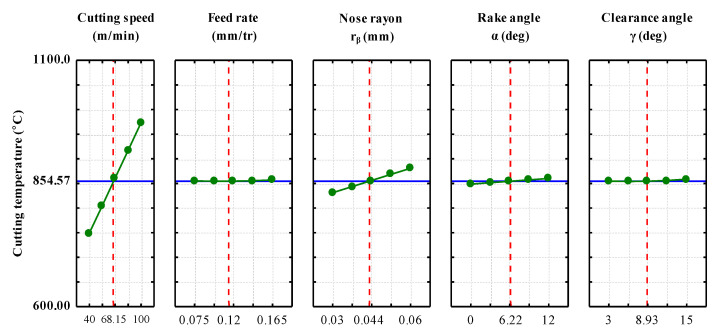
Main effects plots for cutting temperature T_int_ (°C).

Figure 4 shows the direct effect of all factors on the average value of the cutting temperature. The main objective is to show which factors have the maximum effect on the response studied (T_int_). Any increase in the cutting parameters leads to an increase in the cutting temperature. The direct effect of each factor immediately highlights the important factors, which are the cutting speed and the tool nose radius. An increase in the cutting speed leads to an increase in the cutting temperature (30%). A change from a tool with a small nose radius to a tool with a larger nose radius increases the cutting temperature (19%). Factors, such as feed rate, rake angle and clearance angle, appear to have a much smaller effect on the cutting temperature.

#### 3.1.2. Pareto Chart

**Figure 5:**
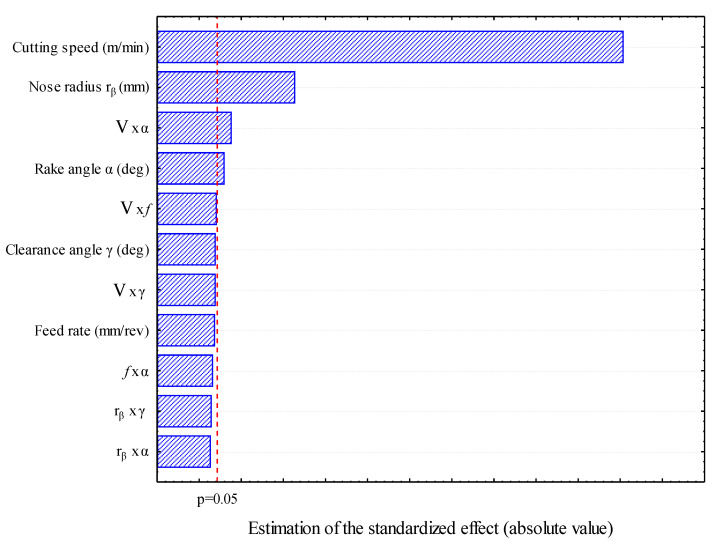
Pareto chart of standardized effects for temperature T_int_ (°C).

The studying of the parameters influence is to determine the combination of factors that would increase the cutting temperature. The Pareto chart allows us to determine the influential factors in order of decreasing contribution. The reading of the Pareto diagram (Figure 5) highlights the predominance of the cutting speed factor on the cutting temperature response. Thus, we can see that, alone, the three factors cutting speed, tool nose radius, rake angle, the interaction between cutting speed and rake angle explain more than 84.66% of the response variability. The contributions of the feed rate and clearance angle are hidden, since their influence is weak. Thus, the factors cutting speed and tool nose radius appear to be those to control in order to increase the cutting temperature. The analysis of the direct effects on the response, their interactions and the order of contribution allowed us to distinguish the large influence of the cutting speed and the tool nose radius on the cutting temperature.

#### 3.1.3. Analysis of Variance

The analysis of variance (ANOVA) allows us to study the main effects of the independent parameters, as well as their interactions, in order to know their combined effects on the dependent response. Based on the significant variables and their interactions, a multiple regression analysis will allow for establishing an empirical model with a coefficient of determination R^2^. In this study, the ANOVA results of the cutting temperature are given in Table 3. The results show that the cutting speed is the factor that represents the largest effect on the cutting temperature variation, which explains 50.39% of the contribution. The second factor that affects the cutting temperature is the effect of the tool nose radius with a contribution of 11.39%, then the interaction between cutting speed and rake angle with a contribution of 3.87%. The final factor is the rake angle effect with a contribution of 3.02%. On the other hand, the other factors and their interactions present very low percentage contributions on the cutting temperature variation.

#### 3.1.4. Regression Model

Regression analysis was used to develop the regression model for the Tint response. The processing of the experimental results obtained in Table 3 allowed the determination of a statistical model, expressing the relationship between the different factors of cutting speed (*V*), feed rate (*f*), tool nose radius (*r_β_*), rake angle (*α*) and clearance angle (*γ*) during orthogonal machining of AMS-6414. In order to establish a model to explain the response, the quality of the model must first be verified. The statistical test that measures the quality of the modeling is the multiple correlation coefficient R^2^, which expresses the ratio of the variance explained by the model to the total variance. To determine which parameters are more influential on the dependent responses in our empirical model, we compared R^2^ by following the stepwise method used manually, which starts from the full model and at each step the associated variable with the largest *p*-value (Table 3) is eliminated from the model. The model coefficient of determination is high and converges to unity indicating good agreement with the experimental results. Table 3 shows the coefficient of determination values (R^2^ ≈ 99.5%) of the proposed model, which indicate good correlation between predicted and experimental machining data. The results compiled in Table 3 show that all variables and their interactions have a significant effect on the dependent variable Tint. This method allowed us to classify according to the fit degree and to choose the required model, which is of the following type:

(18) $$\begin{array}{l} T_{\mathrm{int}}=865.39+222.86_{}V+24.55_{}r_{\beta }+9.42_{}V\alpha +6.68_{}\alpha -5.17_{}Vf+11.70_{}\gamma \\ +4.76_{}V\gamma -11.10_{}f+3.98_{}f_{}\alpha -3.52_{}r_{\beta }\gamma +3.30_{}r_{\beta }\alpha \end{array}$$

The mathematical model analysis allows for defining more precisely the trends as well as the degrees of the different factors influencing on the cutting temperature increase. To this end, analysis of the different factors shows that the greatest influence is reserved for the cutting speed, followed by the tool nose radius and the rake angle, while the feed rate and the clearance angle have a relatively small influence. The validation of the results given by the model consists in examining if the hypotheses retained at the beginning of the experimental design are well verified.

**Figure 6:**
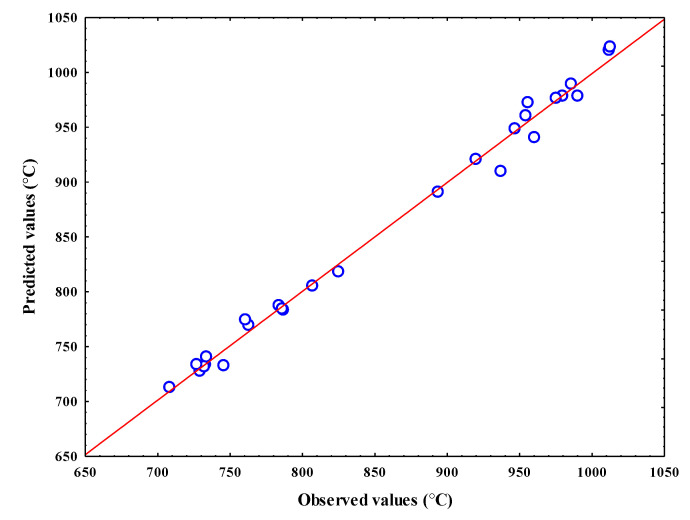
Observed values of the generated temperature against the predicted values with the proposed model.

In our case, all of the mesh nodes of our experimental design are well tested. We have therefore been able to calculate all of the interactions. However, the linearity hypothesis of the response remains to be verified. To do this, if the predicted value distribution is normal to the observed values, the plotted points must align on a line. If an effect does not meet this condition, it means that it deviates from normality, and is therefore likely to be insignificant. The corresponding factor or interaction may therefore be insignificant in this case. The temperature predicted values show that the plotted points are nearly aligned in a straight line (Figure 6). The predicted temperatures are close to the normal line and therefore are normally distributed. The values that deviate from the straight line are due to measurement errors and factors that have been eliminated from the proposed model.

### 3.2. J-C Model Results

#### 3.2.1. Evaluation of Cutting Parameters

**Figure 7:**
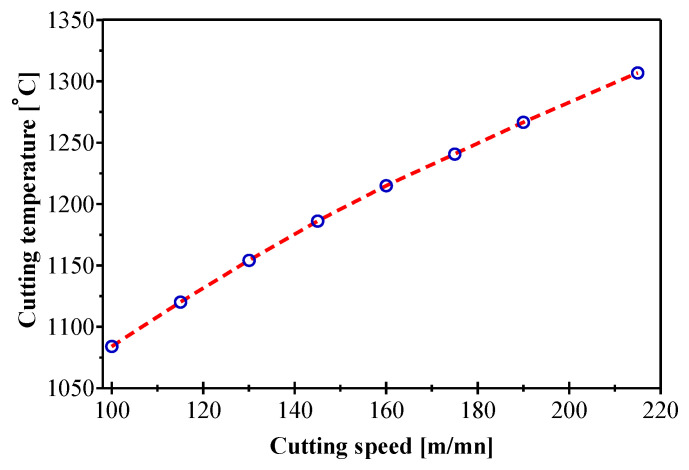
Cutting speed effect on temperature variation for *f* = 0.165 mm.

The cutting temperature predicted by simulation is presented in Figure 7. It is clearly seen that the cutting temperature evolves significantly with the cutting speed, which confirms the experimental results independently of the influence of other cutting parameters (Figure 4). The influence of the cutting speed can be physically visualized and explained from Equation (16) where the generated heat is defined as a function of the flow stress and the chip rate and shear rate are expressed as a function of the cutting speed. When machining AMS-6414, the cut is characterized by a very hot chip flow of red color (tool/chip and tool/workpiece). This quantity of heat is the friction result, intense plastic deformation and shearing. Most of the heat is dissipated through the chip. This prevents the thermal expansion of the workpiece and it will not be thermally affected. In practice, the consequences of the cutting speed influence on the cutting temperature is that an increase in cutting speed from 100 to 220 m/min leads to an increase in the temperature tool/chip by 20.54%. Sometimes, when the speed is high, an elevated temperature in the deformation zone can smooth the progress of the chip flow (ductile material). On the other hand, low cutting speeds can increase the amount of heat transferred to the workpiece.

**Figure 8:**
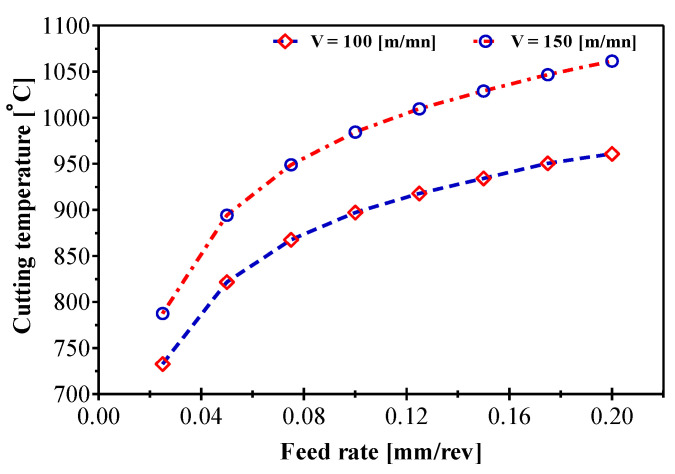
Combined effect of cutting speed and feed rate on the cutting temperature variation during AMS-6414 machining.

Figure 8 shows the simulated cutting temperature evolution for two cutting speeds (100 and 150 m/min) as a function of the feed rate. According to this figure, the temperatures located at the tool/chip contact interface are accentuated by increasing the feed rate. Their maximum values go from more than 732.59 °C to less than 1061.48 °C for feed rates of 0.025 mm/rev and 0.2 mm/rev respectively. The influence of the feed rate is less important than the cutting speed because increasing the feed rate implies an increase in the shear zone width and, consequently, the cutting section or chip area. A large chip cross-section allows for very efficient heat release from the chip. The simulation results agree well with the experimental results; the influence of the feed rate is small but the addition of the interactions between the cutting speed and the feed rate, the rake angle and the feed rate increases the influence intensity of the feed rate. In addition, the simulated heat becomes more propagated through the chip thickness as the feed rate increases and leads to the formation of a larger heat affected zone (HAZ). These high cutting temperatures will have a negative effect on the cutting tool and its service life. This increase in temperature not only affects the cutting tool life, but also the machined surface integrity (residual stress).

#### 3.2.2. Evaluation of the Tool Geometric Parameters

**Figure 9:**
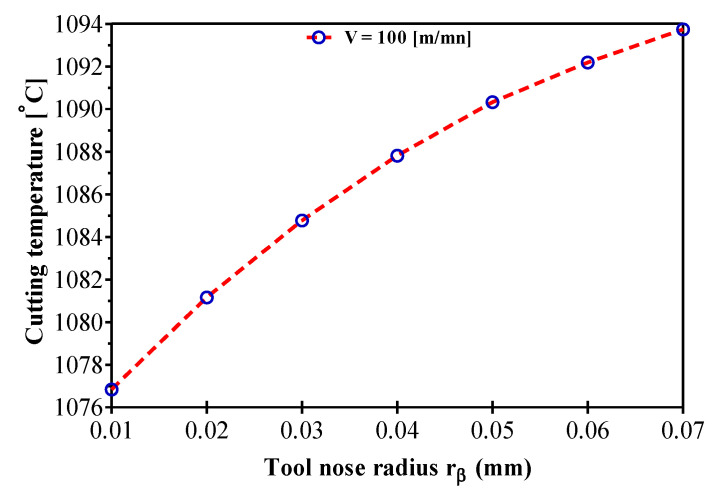
Tool nose radius effect on temperature variation during AMS-6414 orthogonal machining.

We are interested here in understanding the effect of the varying tool nose radius on the thermo-mechanical parameters of the problem. The increase in tool nose radius induces an increase in the cutting temperature, as seen in Figure 9. The cutting temperature increases via the increase of the cutting section area. However, the model results clearly show the influence of the tool nose radius and this increases the cutting temperature. This tendency is due to the apparent friction coefficient variation. The temperature rise induced by the increase in nose radius changes the frictional conditions along the tool cutting face by reducing the shear stress in the secondary shear zone. As a result, BUE becomes more important and friction decreases. This increase in temperature affects the wear of the tool tip.

**Figure 10:**
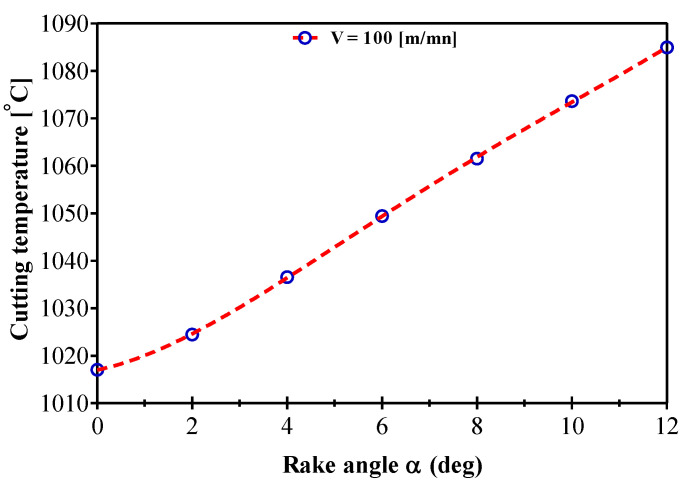
Rake angle effect on temperature variation during AMS-6414 orthogonal machining.

The effect of increasing the rake angle on the cutting temperature at the tool/chip interface was studied to help us to better understand the nature of their influence. From these results, it appears that the positive rake angle increases the cutting temperature by increasing the apparent friction coefficient (Figure 10). The effects of the rake angle on the thermo-mechanical process of chip formation can be explained as follows. The first effect that can be described as geometric is the reduction in the shear angle. This results in an increase in the shear plane length and thus an increase in the cutting forces. On the other hand, the decrease of the rake angle induces an increase of the plastic deformation in the primary shear zone, leading to a higher temperature. An increase in temperature at the tool/chip interface is accompanied by a decrease in the shear stress in the secondary shear zone via thermal softening. Thus, BUE increases as the rake angle decreases, implying a reduction in the friction apparent coefficient that takes into account the full of the sticky-sliding contact. A rake angle close to zero increases chip displacement and can lead to an increase in the quantity of heat transmitted to the part. The clearance angle controls the friction between the workpiece and the tool. A small clearance angle increases the friction between the workpiece and the tool and thus increases the heat generation.

#### 3.2.3. Temperature Distribution at the Tool/Chip Interface

**Figure 11:**
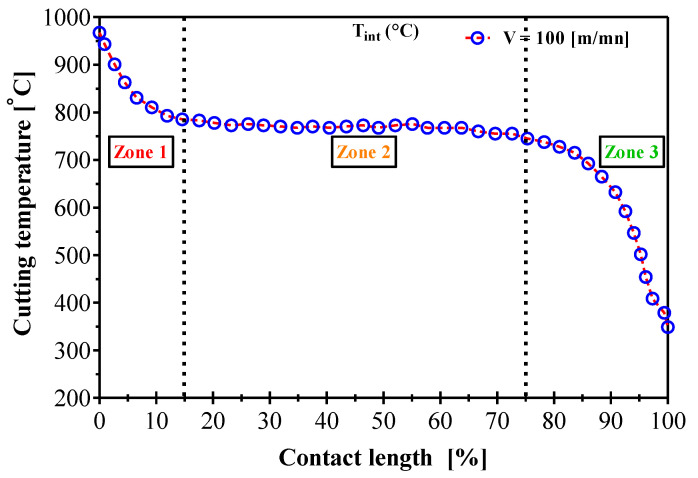
Simulated flash temperature variation in the secondary shear zone during AMS-6414 orthogonal machining.

Figure 11 shows the case of a cutting temperature distribution, assuming that the chip sliding on the tool cutting face is perfect and no wear pattern appears in a case of AMS-6414 machining. The iterative function instantly predicts the flash temperature in the secondary shear zone (SSZ) as a function of the contact length where the friction effect clearly appears. Observation of this curve shows the three zones of cutting temperature variation: the first zone located near the cutting edge in which the cutting temperature variations along the nose radius, the second zone where the cutting temperature is uniform and the third zone (tool/chip separation zone) where the cutting temperature is the same as in the first zone. It can be seen in Figure 11 that the maximum temperature occurred at the beginning of the chip due to its high thermal conductivity which constrains the heat to be conducted to the chip limits. It is also observed that the cutting temperature decreases roughly by 30% on the cutting edge side (zone 1) than on the side of the sliding contact surface of the chip (zone 2) depending on the chip rate reduction. With respect to the local cutting factors, it is noted that the assumption of 100% sliding contact can lead to very high temperatures. This can distort the model results with respect to the thermo-mechanical loading on the tool. When the tool/chip contact is 100% sliding, the heat source is only due to friction. By increasing the BUE, we have a reduction of the heat source and the addition of the heat source by plastic deformation in the secondary shear zone. For these cutting conditions, this effect remains weak. Overall, this study shows the predictive potential of our model and this for a computational time very small to those of finite element simulations. These types of data are used to analyze the cutting tool life via wear models.

#### 3.2.4. Evaluation of Shear Stress

**Figure 12:**
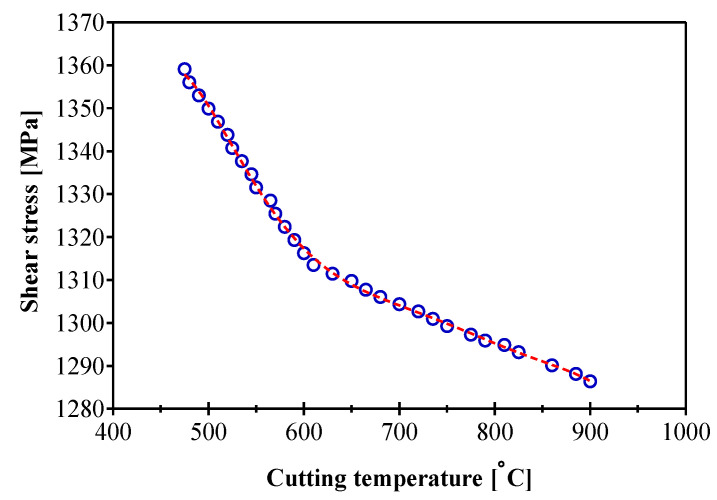
Simulated shear stress variation during AMS-6414 orthogonal machining.

Friction zones are not the only cause of the increase in cutting temperature. Chip formation is identified by four zones, in which the stress modes are different and each of these zones has an influence on the increase in the cutting temperature. When the shear stress at the tool/chip interface is high, the temperature will be transmitted to the tool minor flank next to the cutting edge (tool/workpiece). The temperature effect on the mechanical property can be easily observed in Figure 12 according to the proposed analytical model. This decrease is attributed to the increase in temperature in the contact interfaces and shear bands. The first zone in Figure 11, characterized by higher temperatures than those located on the second zone, has a lower flow stress, which leads to larger and concentrated deformations. The pronounced softening of the material in this first zone leads to the formation of a more concentrated primary shear band. Plastic deformation is high at the material in contact with the tool nose radius and propagates to the free surface creating the primary shear band. The increase in heat in the shear bands results in a more pronounced softening of the material and thus a lower flow stress. This stress decrease is less pronounced (at about 73 MPa) following the increase in cutting temperature of about 425 °C. It can be seen that the cutting temperature combines the effects of the cutting speed and the feed rate. The average stress in the primary zone increases with the increase of the cutting speed for a fixed feed rate. On the other hand, the average stress in the secondary zone decreases with the increasing cutting speed. In addition, the average stresses decreases with the increasing feed rate for a fixed cutting speed. However, the increase in cutting speed has led to an increase in the cutting temperature, which becomes more pronounced for large feeds. This change in the average value of the shear stress was noticed by varying the feed rate. This decrease is explained by the material removal rate due to the variation of the feed rate in the range of speeds studied. We also add that, regardless of the defined feed rate, the shear stress decreases slightly with increasing cutting speed. It was therefore concluded that the shear stress limit in the Zorev model should be used with caution to obtain an accurate machining temperature.

### 3.3. Response Surface

**Figure 13:**
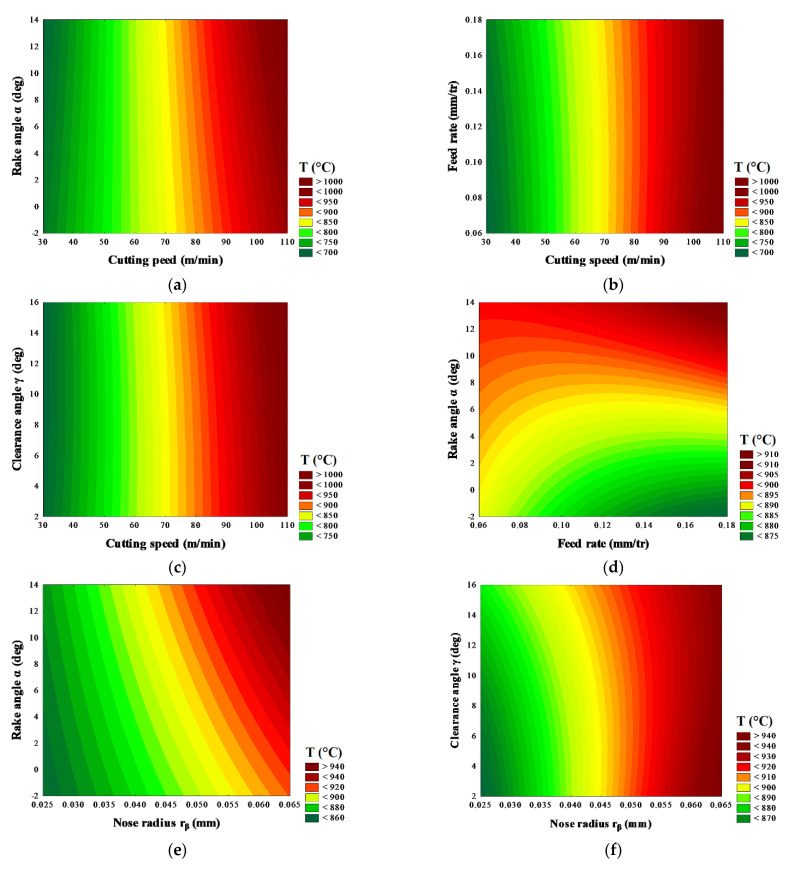
Cutting temperature variation as a function of different factor interactions: (**a**) cutting speed and rake angle; (**b**) cutting speed and feed rate; (**c**) cutting speed and clearance angle; (**d**) feed rate and rake angle; (**e**) tool nose radius and rake angle; (**f**) tool nose radius and clearance angle.

The graphical representation of the regression model equation allows us to illustrate the variations in the response and eventually to identify areas of the experimental field in which the cutting temperature is maximum or minimum. The response surfaces (Figure 13) concretize the cutting temperature variation as a function of the various factors of cutting speed (*V*), feed rate (*f*), tool nose radius (*r_β_*), rake angle (*α*) and clearance angle (*γ*) during AMS-6414 orthogonal machining. From Figure 13, it can be seen that an increase in the cutting temperature takes place for the different cutting conditions. It is observed that the predicted cutting temperature increased with the increase in cutting speed and the bigger nose radius resulted in higher machining temperature during orthogonal cutting. The predicted maximum tool temperatures on the cutting face were similar for all feed rates considered, while the temperature at the tool tip increased with the increasing tool nose radius. It was also found that the effect of the clearance angle on the temperature profile was much less significant than that of the cutting speed. From Figure 13a–c, the cutting speed has a significant effect on the increase in the cutting temperature, regardless of the rake angle, feed rate and clearance angle used. On the other hand, for the cutting speeds range studied, an increase in the cutting temperature is noticed as a result of the increase in tool nose radius and rake angle. The heat amount is about 12.5% compared to the defined tool nose radius, regardless of the cutting speed. In this study, the maximum is given by a cutting speed (*V* = 100 m/min), feed rate (*f* = 0.165 mm/rev), rake angle (*α* = 12°) and for a specific tool geometry (*r_β_* = 0.06 mm). The main conclusion from these curves is that there are combinations between the different factors (*V.f, V.α, V.γ, f.α, r_β_.α, r_β_.γ*) for which the cutting temperature is low. In the considered experimental field, the response surfaces emphasize the major importance of the rake angle factor on the level of cutting temperature reached by the cutting speed. The feed rate factor also shows a non-negligible role in obtaining high cutting temperatures, but to a less significant degree compared to the interaction between the cutting speed and rake angle, which can also be explained by the relatively small feed rate range explored. This leads us to conclude that the cutting speed factor is of paramount importance in cutting temperature management.

## 4. Discussion

The study of the cutting temperature carried out in this work allows us to better choose the parameters of orthogonal machining with the aim of achieving better stability of the machining. The cutting temperature is one of the most critical quantities of the cutting process, because a high cutting temperature leads to inaccuracies in the part’s dimensions, the phase transformation and, above all, the creation of residual stresses. The analysis showed that the cutting speed parameter has a direct effect on the increase of the cutting temperature. The maximum cutting temperature was found to be at the tool/chip interface, around the cutting edge. In addition, it is noted that the location at which the maximum temperature occurs depends primarily on the workpiece material conductivity and especially the cutting speed. This effect is significant if the selected cutting angle is equal to a critical rake angle. Similar to the experimental tests, the strong dependence of the cutting temperature on the cutting speed has been numerically reproduced. From the experimental/simulation correlation, the temperatures determined by the proposed cutting parameters are close to the experimental ones. On the other hand, for higher feed rates, the comparison with the experimental results underlines a significant overestimation of the average value of this temperature for all of the cutting speeds studied. To validate the model, seven tests were performed for a fixed cutting speed of 100 m/min where a comparative study between the experimental, analytical and regression function is conducted in Table 4.

**Figure 14:**
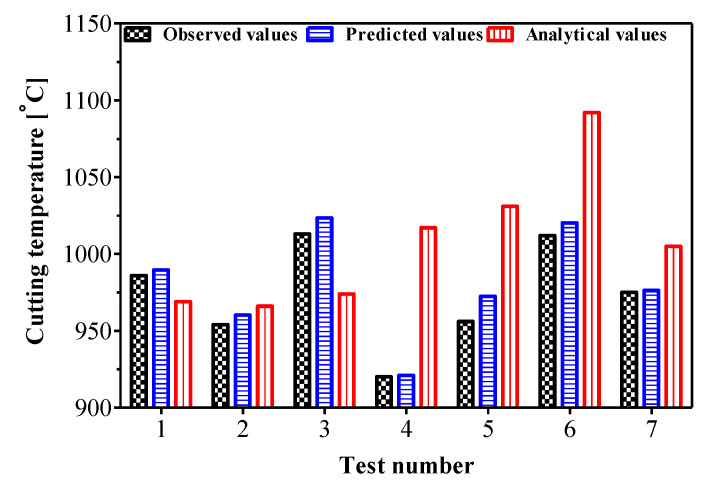
Cutting temperature validation for the different cutting conditions studied.

The comparison between the three responses to temperature in the secondary shear zone is plotted in Figure 14. Good agreements were observed between the predicted temperatures and the measured values with less than a 10% error. The regression and the J-C model offer an advantage in terms of fast prediction due to their mathematical formulation. In addition, the modeling of the orthogonal cut using the J-C plasticity model gave an interesting level of correlation with the experimental tests in terms of cutting temperature.

The calculated deviations for the different cutting conditions tested are relatively acceptable. This justifies that the modifications proposed in the analytical model that incorporated the moving source theory to express the maximum temperature for an orthogonal cutting application led to the reliability of the J-C plasticity model modeling of the orthogonal machining process. On the other hand, the J-C model reproduced the fact that the temperature is higher on the cutting edge side (zone 1, Figure 11) than on the free surface side of the chip (zone 3). The deviation between the predicted temperatures and the analytical values can be larger (±5.54%) than the measured values due to the model non-linearity or the parameter needed for some phenomena and physical parameters that affect the results.

**Figure 15:**
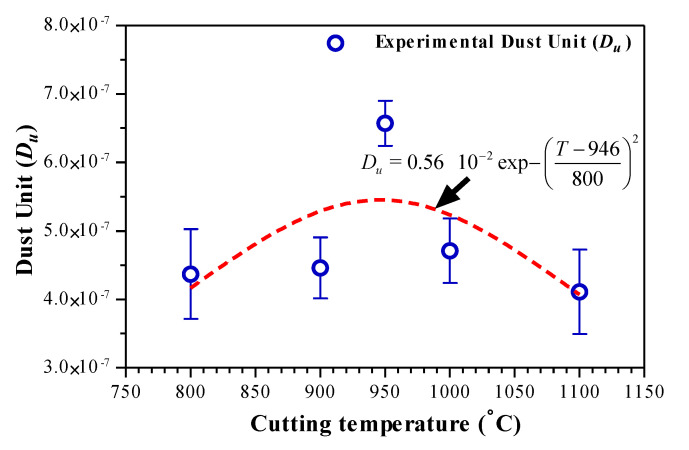
Cutting temperature effect on dust emission during AMS-6414 orthogonal cutting.

The dust emission modeling proposed by Khettabi et al. is based on the energetic approach, combined with the macroscopic friction (tool/chip), micro-friction and plastic deformation of materials [22]. The dust generation can indicate different phenomena of fine and ultrafine particle generation during the cutting process related to the heat source or temperature in the orthogonal machining. From this analysis, we confirm that the dust should be presented as a function of the cutting temperature and not as a function of the cutting speed. This is because, to generate these dusts and the particles that leave the material, it is necessary to provide sufficient thermo-mechanical energy to break the bonds. The particle size depends on the heat source that activates their detachment energy states. Ultrafine particles have a smaller energy state than fine particles. Measurements of Dust Unit (*D_U_*) are defined as the ratio between the dust mass and the chip’s mass removed from the AMS-6414 orthogonal machining. The results obtained are shown in Figure 15. The experimental results show that the dust emission follows an exponential law as a function of the cutting temperature increasing (see the equation in Figure 15). In this case, the increase in temperature activates the energetic state of these particles and the dust generation increases. Thus, the increase in temperature in the cutting zone allows for the ultrafine particle separation. However, the dust generation decreases above a critical temperature (950 °C). This means that they have a smaller amount of excess energy, which directly means a reduction in the micron-sized dust generation.

The effect that we have just described is to analyze in general the mode of dust emission as a function of the cutting temperature variation. We are looking for the cutting parameters for which we obtain the minimum emission or we identify the cutting temperature as the key response to limit the dust generation during the orthogonal machining. On the other hand, the measurement of temperature will be difficult in the case of some cutting processes. Despite this, increasing the cutting speed leads to an increase in temperature. This can be attributed to the use of cutting speed values to estimate the critical temperature. In addition, this confirms the importance of the optimization of the cutting temperature by RSM that allows for identifying the combination of cutting conditions and tool geometry to obtain the minimum dust emission despite their randomness in the machining case. Table 5 illustrates the RSM optimization results for the cutting temperature:

The effective strategy to limit dust emissions at the source is to avoid the critical temperature zone that corresponds to the cutting conditions values and the tool geometry predicted in Table 5. To this end, the two-sided values on the left and right of Figure 15 can be seen as combinations to limit dust emissions at the source.

## 5. Conclusions

Johnson-Cook’s model, which is considered as the most fundamental approach in analytical modeling of the machining process, was generalized to the dust generation evaluation. The model was successfully used in predicting the cutting temperature in machining and good agreement has been found between the prediction and experiments, with a confidence level equal to 95%. The main contributions achieved in the present study can be summarized as:

✓ The experimental temperature correlates well with the predicted temperature with less than 10% uncertainty. This confirms that the presented temperature model can be used for the prediction of the cutting temperature of metallic materials;
✓ The identification of three factors controlling the temperature variation during orthogonal machining: cutting speed, tool nose radius, rake angle and the interaction between cutting speed and rake angle;
✓ The establishment of a relationship between temperature variation and dust generation;
✓ The dust generation which is the thermo-mechanical origin, can be quantified by the cutting temperatures during orthogonal machining and it is closely related to the heat source generated in the SSZ.

The conclusion from the present study is that the reduction of dust generation is possible at the source under any heating of the workpiece. The proposed approaches can be used in the optimization of the cutting conditions in order to control the dust generation on the machined parts. This work is interesting all industrial applications, because, currently, the laws on health and safety at work are evolving, following the increasing frequency of occupational diseases compensated by health insurance. In addition, the industrial interest of this mathematical model is considerable because they allow the determination of optimal cutting conditions and give valuable information on the cutting process.

## Figures and Tables

**Table 1:** Chemical and physical parameters of the machined parts.

AMS-6414	C	Mn	Cr	Ni	Mo	Cu	Si	P	S	Fe
Chemical compositions (%)	0.4	0.7	0.76	1.76	0.24	0.1	0.27	0.004	0.001	Balance
	**ρ_w_ (kg/m^3^)**	**K_w_ (W/m°C)**	**C_w_ (J/kg°C)**	**T_m_ (°C)**	**ρ_t_ (kg/m^3^)**	**K_t_ (W/m°C)**	**C_t_ (J/kg°C)**			
Physical properties	7850	44.5	475	1427	14320	68.1	280			
	**A**	**B**	**C**	**n**	**m**	**έ_0_**	**T_0_**	**T_m_**		
J-C Constant	792	510	0.014	0.26	1.03	0.001	25	1520		

**Table 2:** Machining parameters and their levels.

Level	*V* (m/min)	*f* (mm/rev)	*r_β_* (mm)	*α* (deg)	*γ* (deg)
1	40	0.075	0.03	0	3
2	60	0.105	0.04	4	7
3	80	0.135	0.05	8	11
4	100	0.165	0.06	12	15

**Table 3:** ANOVA results of cutting temperature variance.

ANOVA: R-sqr = 99.489%; R-adj = 99.114%; MS Residual = 106.4216 DV						
	Adj-SS	Df	Adj-MS	F-Value	*p*-Value	P (%)
**Cutting speed (m/mn)**	**270,227.7**	**1**	**270,227.7**	**2539.220**	**0.000000**	**50.39%**
Feed rate (mm/rev)	381.1	1	381.1	3.581	0.077909	1.89%
**Nose radius *r_β_* (mm)**	**13,828.4**	**1**	**13,828.4**	**129.940**	**0.000000**	**11.39%**
**Rake angle *α* (deg)**	**971.2**	**1**	**971.2**	**9.126**	**0.008599**	**3.02%**
Clearance angle *γ* (deg)	415.3	1	415.3	3.903	0.066908	1.98%
*V* × *f*	469.6	1	469.6	4.412	0.052999	2.10%
***V* × *α***	**1594.4**	**1**	**1594.4**	**14.982**	**0.001509**	**3.87%**
*V* × *γ*	412.5	1	412.5	3.876	0.067750	1.97%
*f* × *α*	287.3	1	287.3	2.700	0.121137	1.64%
*r_β_* × *α*	205.7	1	205.7	1.933	0.184722	1.39%
*r_β_* × *γ*	235.4	1	235.4	2.212	0.157665	1.49%
Error	1596.3	15	106.4			
Total SS	312,181.2	26				

**Table 4:** Cutting temperature validation results for AMS-6414 orthogonal machining at a cutting speed of 100 m/min.

Test Number	*f* (mm/tr)	*r_β_* (mm)	*α* (deg)	*γ* (deg)	Observed Values (°C)	Predicted Values (°C)	Analytical Values (°C)
1	0.075	0.06	0	3	986	989.53	969
2	0.075	0.03	0	15	954	960.27	966
3	0.075	0.06	12	15	1013	1023.52	974
4	0.165	0.03	0	3	920	921.01	1017
5	0.165	0.06	0	11	956	972.47	1031
6	0.165	0.06	12	3	1012	1020.20	1092
7	0.165	0.03	12	15	975	976.25	1005

**Table 5:** Predicted response at each factor in the regression model for a maximum value of the cutting temperature.

*V* (m/min)	*f* (mm/tr)	*r_β_* (mm)	*α* (deg)	*γ* (deg)	T_int_ (°C)
100	0.165	0.06	12	15	1022.98

## Data Availability

Not applicable.
